# Patterns and predictors of infant and young child feeding practices among mothers of children aged 6–23 months in Plateau State, Nigeria: a mixed-methods comparison of nutrition intervention beneficiaries and non-beneficiaries

**DOI:** 10.3389/fnut.2026.1951497

**Published:** 2026-09-30

**Authors:** Demilade Osoteku, Abdulateef Salisu, Muyi Aina, Precious Uahomo, Majeed Adisa, Michael Obiabo, Hajara Nakkudu, Mercy Yesin, Kakagoni Yusuf, Makrop Dangok, Zakariyau Haruna, Michael Okoh, Precious Otono, Tobiloba Adaramati, Oluwabori Samuel, Haishat Olufadi-Ahmed, Valentine Amasiatu, Eric Aigbogun, Uchenna Igbokwe

**Affiliations:** 1 Solina Centre for International Development and Research (SCIDaR), Abuja, Nigeria; 2 National Primary Health Care Development Agency (NPHCDA), Abuja, Nigeria

**Keywords:** Accelerating Nutrition Results in Nigeria (ANRiN), complementary feeding, infant and young child feeding (IYCF), knowledge attitudes and practices (KAP), maternal nutrition, Nigeria, Plateau state, sociodemographic determinants

## Abstract

**Background:**

Nigeria ranks second globally in the burden of stunted children, with 40% of children under five affected and poor infant and young child feeding (IYCF) practices as a primary contributor. This study assessed mothers' knowledge, attitudes, and feeding practices related to IYCF in Plateau State, Nigeria, comparing beneficiaries and non-beneficiaries of the Accelerating Nutrition Results in Nigeria (ANRiN) project, and identifying sociodemographic predictors associated with these outcomes.

**Methods:**

A mixed-methods cross-sectional design was employed. A total of 503 mothers of children aged 6–23 months completed structured questionnaires for quantitative analysis, while 8 focus group discussions and 12 key informant interviews provided qualitative data. Knowledge, attitude, and practice scores were computed with categorization (adequate/inadequate, positive/negative, good/poor). Data were analyzed using chi-square tests, independent *t*-tests, and multivariable logistic regression stratified by beneficiary status.

**Results:**

The overall prevalence of adequate knowledge was 21.1%, positive attitudes 91.4%, and good feeding practices 55.7%. Beneficiaries demonstrated significantly better practice scores than non-beneficiaries (mean 6.95 ± 1.42 vs. 6.34 ± 1.57, *p* < 0.001), while non-beneficiaries showed slightly higher attitude scores (3.68 ± 0.64 vs. 3.52 ± 0.77, *p* = 0.012). No significant difference in knowledge scores was observed. Maternal tertiary education was significantly associated with adequate knowledge in both groups (*p* < 0.01), while healthcare provider information was the strongest predictor of knowledge and attitudes among beneficiaries (*p* < 0.001 and *p* = 0.019, respectively). Qualitative findings identified poverty, food insecurity, and cultural norms as barriers to applying knowledge, while hands-on food demonstrations improved practical skills.

**Conclusion:**

The study highlights a persistent knowledge-practice gap and demonstrates that ANRiN participation is associated with improved feeding practices. The distinct predictors between beneficiaries and non-beneficiaries suggest that tailored interventions accounting for these differential factors could strengthen IYCF program effectiveness in Nigeria.

## 1 Introduction

Malnutrition remains a major public health challenge in low- and middle-income countries (LMICs), particularly among infants and young children, for whom adequate nutrition is essential for survival, growth, and cognitive development (1). In Nigeria, child malnutrition remains widespread, with the 2024 Nigeria Demographic and Health Survey (NDHS) reporting that 40% of children under five are stunted, 8% are wasted, 27% are underweight, and 1% are overweight (2). Compared with the 2018 NDHS estimates of 37% stunting, 7% wasting, 22% underweight, and 2% overweight, these figures indicate that substantial nutritional challenges persist (1, 3). Nigeria also has one of the largest populations of stunted children globally, with approximately 32% of children under five affected and an estimated two million children experiencing severe acute malnutrition (SAM), of whom only about 20% receive treatment (4).

Infant and young child feeding (IYCF) is an important determinant of child nutritional status and survival. Optimal IYCF encompasses early initiation of breastfeeding, exclusive breastfeeding (EBF) during the first 6 months, timely introduction of adequate and appropriate complementary foods from 6 months, continued breastfeeding, dietary diversity, appropriate meal frequency, and adequate intake of essential micronutrients (5–8). The World Health Organization (WHO) and United Nations Children's Fund (UNICEF) recommend initiation of breastfeeding within the first hour of birth, EBF for the first 6 months, followed by continued breastfeeding alongside nutritionally adequate and diverse complementary foods (6). These practices are important not only for preventing undernutrition but also for reducing childhood morbidity and supporting healthy growth and development.

Despite these recommendations, optimal IYCF practices remain suboptimal in Nigeria. For example, EBF has shown limited improvement over time, increasing from 17% in 2013 to 23% in 2017 and 29% in 2018 among children under 6 months (9–12). However, focusing on EBF alone provides only a partial picture of infant and young child nutrition, as feeding practices after 6 months are equally important for meeting children's increasing nutritional requirements. Evidence from Nigeria and other LMICs indicates persistent challenges with timely complementary feeding, dietary diversity, meal frequency, and access to nutrient-rich foods (2, 6, 13, 14). These challenges contribute to inadequate nutrient intake and increase vulnerability to growth faltering and other forms of malnutrition.

The burden of child malnutrition also varies substantially across Nigeria's geopolitical zones. The North West (53.2%) and North East (51.8%) recorded the highest prevalence of stunting, compared with South-South (17.9%) and South East (20.6%). Underweight prevalence was similarly higher in the North East (33.4%) and North West (33.8%) than in the South West (21.8%) and South-South (16.8%) (2). Within the North Central zone, Plateau State recorded a stunting prevalence of 46.4%, followed by Niger (43.9%) and Kwara (40.8%), while underweight prevalence was 22.8% in Plateau, 23.7% in Niger, and 24.5% in Kwara (2). These disparities highlight the uneven distribution of nutritional challenges across the country and the need for context-specific interventions (2, 15).

Mothers and other caregivers play a central role in determining what, when, and how children are fed. Their knowledge and attitudes toward recommended feeding practices can influence adoption of appropriate behaviors, but knowledge alone may not translate into practice where caregivers face financial constraints, limited access to nutritious foods and health services, cultural barriers, or inadequate household support (16, 17). Evidence from nutrition interventions suggests that behavior change communication, counseling, micronutrient interventions, and community-based support can improve caregivers' knowledge and feeding practices, although the magnitude and sustainability of these effects vary across settings (18–23).

Nigeria has implemented several policies and programmes to address these challenges. The National Policy on IYCF promotes optimal breastfeeding and complementary feeding practices, including early initiation of breastfeeding and EBF during the first 6 months of life (24). However, persistent gaps between recommended practices and actual feeding behaviors indicate the need for effective implementation of community-based nutrition interventions. The World Bank-funded Accelerating Nutrition Results in Nigeria (ANRiN) project was introduced to strengthen nutrition services for pregnant and lactating women, adolescent girls, and children under five, particularly in high-burden settings (25). The programme uses community-based platforms, including community health workers (CHWs), to deliver nutrition interventions and social and behavior change communication (SBCC) aimed at improving maternal, infant, and young child nutrition (MIYCN) practices (26, 27).

Evidence from ANRiN and similar interventions suggests potential benefits for maternal and child nutrition outcomes. For example, a study among ANRiN beneficiaries in Kwara State found significantly higher knowledge and attitude scores compared with non-beneficiaries, while some sociodemographic characteristics were associated with improved feeding practices (28). Evidence from other community-based interventions also indicates that counseling, behavior change communication, and locally adapted nutrition education can improve caregivers' knowledge and IYCF practices (18, 20–22, 28). However, findings from one setting may not necessarily be generalizable to others because implementation context, service access, household characteristics, and cultural practices vary across states.

Plateau State is one of the settings in which ANRiN has been implemented to strengthen community-based nutrition services and improve IYCF practices. Despite the implementation of the programme, there is limited evidence on whether exposure to ANRiN is associated with differences in mothers‘ and caregivers' IYCF knowledge, attitudes, and feeding practices in the state. Existing evidence has largely focused on specific components of IYCF or other programme settings, leaving an important gap in understanding the broader relationship between participation in a large-scale community-based nutrition intervention and multiple dimensions of IYCF (19, 23, 28).

This study therefore compares the knowledge, attitudes, and feeding practices of mothers and caregivers of infants and young children who are beneficiaries and non-beneficiaries of the ANRiN intervention in Plateau State. Specifically, the study aims to (i) assess differences in IYCF knowledge, attitudes, and practices between beneficiaries and non-beneficiaries, and (ii) determine the sociodemographic factors associated with these outcomes, with exposure to ANRiN considered as a key explanatory factor. We hypothesized that exposure to ANRiN would be positively associated with higher IYCF knowledge, more favorable attitudes, and improved feeding practices after accounting for relevant sociodemographic characteristics. By examining multiple dimensions of IYCF rather than exclusive breastfeeding alone, this study provides evidence on the relationship between large-scale community-based nutrition programming and maternal and caregiver feeding behaviors in Plateau State, Nigeria.

## 2 Methods

### 2.1 Study design

This study employed a mixed-methods cross-sectional study design (29).

### 2.2 Study area

This study was conducted in Plateau State, Nigeria, where the ANRiN project was actively implemented. Plateau State is located in the north-central geopolitical zone of Nigeria. The state has 17 local government areas and an estimated population of over 3.5 million people (30). The state is known for its diverse ethnic groups, cultural practices, and agricultural activities, which may influence infant and young child feeding practices. The study was carried out in Mangu and Shendam Local Government Areas (LGAs) of the state (Figure 1), which were selected as representative areas of the state's nutritional intervention coverage.

**Figure 1:**
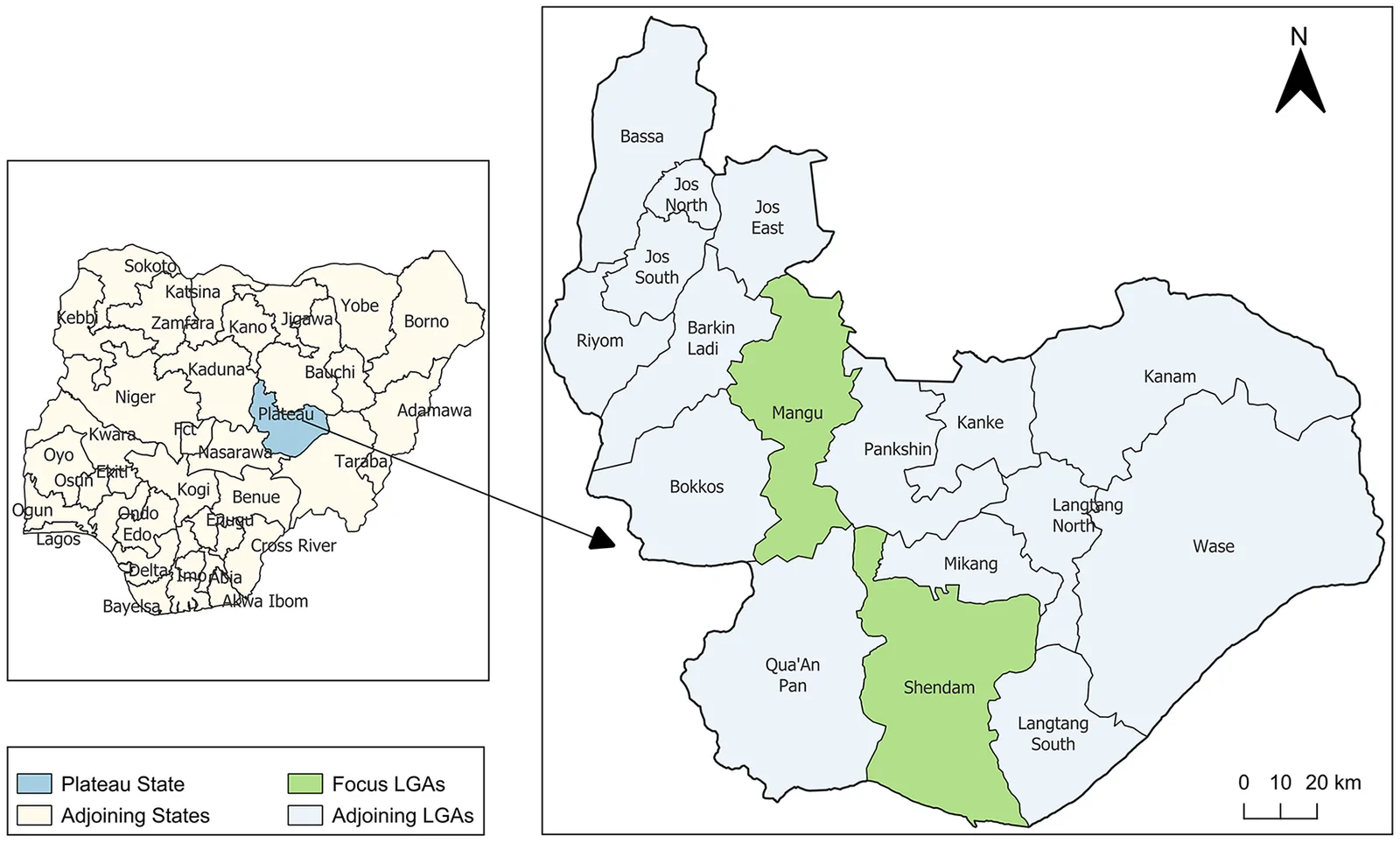
Map of Plateau State showing study LGAs.

### 2.3 Sample size determination

The sample size for the quantitative component was calculated using the Cochran formula for cross-sectional studies with populations above 10,000 people: n = (Z^2^pq)/e^2^ (31), where n represents the required sample size, Z is the standard normal deviation set at 1.96 for a 95% confidence level, *p* is the estimated proportion of mothers with adequate knowledge of infant and young child feeding, and d is the desired level of precision set at 0.05. A value of *p* = 0.5 was adopted to maximize sample size precision because no prior data were available on the prevalence of adequate maternal IYCF knowledge in the study population. The minimum sample size calculated was 384; after accounting for a 10% non-response rate, the final minimum sample size was 422 participants. For the qualitative component, a purposive sample of 8 FGDs (4 among beneficiaries and 4 among non-beneficiaries, each with 8 participants) and 12 KIIs with stakeholders was determined based on thematic saturation (32), yielding a total of 64 mothers and 12 stakeholders.

### 2.4 Sampling and participant recruitment

A multistage sampling technique was used to recruit participants for the quantitative survey. Data collection was conducted between July and August 2024. In stage 1, out of the 17 LGAs in the state, 2 LGAs (Mangu and Shendam) were selected using simple random sampling (SRS) without replacement. In stage 2, out of 11 wards and 10 wards in Mangu and Shendam LGAs, respectively, 2 wards were randomly selected from each LGA. Within each selected ward, 2 communities were randomly selected per ward in each LGA. Random selection at each stage was performed using a computer-generated random number list. Due to population differences across LGAs, quota allocation was applied based on population size: Mangu LGA was allocated 60% (253 participants) of the total sample size, and Shendam LGA was allocated 40% (169 participants). In stage 3, within each community, households with mothers of children aged 6–23 months were identified through house-to-house enumeration conducted by trained community health volunteers. Households were selected using systematic random sampling from the enumeration list, with a random starting point and a fixed sampling interval. One eligible mother was recruited per household. Households were randomly selected until the predetermined quota for each LGA was reached. This approach resulted in sampling multiple households across the communities to achieve the target sample size.

Eligible participants were mothers/caregivers of children aged 6–23 months who had resided in the study communities for at least 6 months. Exclusion criteria included mothers whose children had severe acute illness requiring hospitalization at the time of data collection and mothers who were unable to provide informed consent.

Beneficiary status was not a sampling criterion. After enrolment, participants were categorized based on their exposure to the ANRiN intervention. Beneficiaries were defined as mothers who had actively received ANRiN services such as nutrition counseling, growth monitoring, or micronutrient supplementation, through accredited CHWs or designated service delivery points. Non-beneficiaries were mothers residing in the same LGAs who had not received any ANRiN-provided services. Non-beneficiaries were sampled from the same LGAs as beneficiaries to control for geographic, socioeconomic, and health system factors that could independently influence IYCF KAP. This approach enhances comparability by ensuring both groups were exposed to similar contextual conditions, aside from direct ANRiN service receipt.

For the qualitative component, participants were purposively selected from the same communities. FGD participants were recruited among mothers (both beneficiaries and non-beneficiaries) from randomly selected communities within the study LGAs. The KII participants were purposively selected based on their roles and included members of the ANRiN Project Implementation Units (PIUs), Nutrition Officers, and CHWs across the two study LGAs.

### 2.5 Quantitative data collection instrument and study measures

The data collection tool was developed on KoboCollect because of its ability to gather geospatial data, ensuring that the data were collected at verifiable and precise locations. The data collection was performed using interview-administered questionnaires, facilitated by trained Community Health Volunteers selected for their experience in survey administration. For supervision and quality control, authors PU, OS, MO, and MY provided field-level oversight and supported the process through backend data validation via KoboCollect. This collaboration facilitated ease of access to research respondents and ensured their commitment to completing the questions. The staff directly interfaced with the research assistants, thereby minimizing data collection errors and biases.

Data collectors underwent a 5-day intensive training covering: (i) study objectives and ethical considerations, (ii) questionnaire administration and probing techniques, (iii) use of KoboCollect for data collection, (iv) role-playing and field practice, and (v) quality assurance procedures. Training was conducted by experienced researchers from the SCIDaR Knowledge Management and Learning (KML) Unit. Field supervisors conducted daily debriefing sessions and reviewed completed questionnaires for completeness and consistency.

The Food and Agriculture Organization (FAO) publication guidelines for assessing nutrition-related knowledge, attitudes and practices (33), also known as the KAP manual, and the questionnaire from Bimpong et al. (34) were adopted to develop the questionnaire for this study. The questionnaire collected information on the sociodemographic characteristics of mothers and infants, including age, sex, marital status, educational level of both the caregiver and partner, religion, employment status, and monthly income brackets. Household characteristics were also captured, such as the number of children, the sex and age of the index child, and household size. Given the potential influence of large-scale nutritional programs on IYCF outcomes, participants' status as beneficiaries or non-beneficiaries of the ANRiN project was recorded as a key covariate.

Knowledge assessment consisted of 11 items evaluating mothers‘ understanding of key infant and young child feeding concepts, including the timing of breastfeeding initiation, duration and benefits of EBF, timing of complementary feeding introduction, dietary diversity, and the role of micronutrients such as vitamin A, iron, and iodine. Each correct response was scored “1” and incorrect or “don't know” responses were scored “0.” The total knowledge score for each respondent was computed as the sum of correct responses (range: 0–11). Attitude assessment included four items exploring participants' beliefs and perceptions regarding breastfeeding vs. formula feeding, the appropriate age for introducing solid foods, and the importance of balanced nutrition for child growth and development. Responses were scored on a 5-point Likert scale, ranging from “strongly disagree” [1] to “strongly agree” [5]. Practice assessment measured mothers‘ adherence to WHO-recommended IYCF guidelines, including exclusive breastfeeding during the first 6 months, continued breastfeeding beyond 6 months, meal frequency, dietary diversity, and hygiene practices. Mothers' responses were used to compute complementary feeding indicators such as minimum meal frequency, minimum dietary diversity, and minimum acceptable diet, following WHO definitions (13, 14). A composite practice score was derived (range: 0–10). The sources of IYCF information were also investigated.

The scoring algorithm for knowledge was: total knowledge score = sum of correct responses (range: 0–11). Adequate knowledge was defined as a score ≥70% (≥7.7 out of 11), while inadequate knowledge was < 70% (< 7.7). For attitude, total score = sum of Likert responses (range: 4–20), with positive attitude defined as ≥70% (≥14 out of 20) and negative attitude as < 70% (< 14). For practice, total score = sum of correct responses (range: 0–10), with good practice defined as ≥70% (≥7 out of 10) and poor practice as < 70% (< 7). Mothers' knowledge and practice were categorized as “adequate” (≥70%) or “inadequate” (< 70%), and attitudes were classified as “positive” (≥70%) or “negative” (< 70%) based on thresholds commonly applied in similar KAP studies (33–35), and validated during pretesting.

### 2.6 Qualitative data collection

To complement the quantitative data, qualitative methods were employed to gain deeper insights into the factors influencing infant and young child feeding attitudes and practices. FGDs were conducted among purposively selected mothers (both beneficiaries and non-beneficiaries) from randomly selected communities. In addition, key informant interviews (KIIs) were held with stakeholders, including members of the ANRiN Project Implementation Units, Nutrition Officers, and Community Health Workers, across the two study LGAs. These qualitative engagements explored perceptions of the effectiveness of ANRiN's food demonstrations, the influence of community-based counseling on practical feeding skills, and barriers to translating knowledge into sustained feeding practices.

FGD guides explored: (i) current feeding practices and challenges, (ii) sources of nutrition information, (iii) perceptions of ANRiN program activities, (iv) barriers to practicing recommended feeding, and (v) suggestions for program improvement. KII guides examined: (i) program implementation experiences, (ii) challenges in service delivery, (iii) perceptions of program effectiveness, and (iv) recommendations for program enhancement. The complete guides are provided as Supplementary File S1.

Trained data collectors fluent in Hausa, the predominant local language, identified and recruited eligible participants in each study community. Each FGD session comprised eight participants and was moderated by experienced qualitative researchers under the supervision of senior field coordinators to ensure methodological consistency and adherence to the discussion guides. A total of eight FGDs were conducted (four among beneficiaries and four among non-beneficiaries), alongside 12 KIIs with key stakeholders, resulting in a total of 76 qualitative participants (64 mothers and 12 stakeholders). All interviews and discussions were conducted in participants' preferred language, with informed consent obtained prior to audio recording. Interviews continued until thematic saturation was reached, with no new themes emerging from subsequent discussions.

### 2.7 Validity and reliability of the data collection tool

The questionnaire used in the study was evaluated for both validity and reliability. Face validity was established through review by a panel of research experts and academics in the Knowledge SCIDaR KML Unit, who assessed each item for clarity, relevance, and appropriateness. Their feedback informed revisions that improved the precision and contextual suitability of the questions. The instrument was subsequently pre-tested among a sample of 32 mothers/caregivers in a community outside the study area to identify potential ambiguities and ensure cultural relevance, after which final adjustments were made prior to full deployment. These 32 participants were not included in the final analytic sample.

To assess internal consistency, Cronbach's alpha coefficients were calculated for each of the KAP domains. The Knowledge scale demonstrated acceptable reliability (α = 0.78), the Attitude scale showed good internal consistency (α = 0.82), and the Practice scale yielded an alpha of 0.75, indicating satisfactory reliability. These values confirm that the instrument was both valid and reliable for measuring KAP related to infant and young child feeding in the study population.

For the qualitative component, trustworthiness was ensured through triangulation of data sources (mothers, health workers, and program staff), investigator triangulation (multiple researchers involved in analysis), and member checking (participants reviewed summaries of findings to confirm accuracy). Detailed field notes and an audit trail were maintained to enhance dependability and confirmability.

### 2.8 Data cleaning and statistical analysis

#### 2.8.1 Quantitative data analysis

Quantitative data cleaning involved excluding 3 records with missing or inconsistent responses from the initial 506 collected, resulting in a final analytic sample of 503 mothers (187 non-beneficiaries and 316 beneficiaries). Data were first prepared in Microsoft Excel 2016 and then analyzed using IBM SPSS Version 26. Descriptive analyses were conducted to summarize participants' sociodemographic characteristics (such as age, marital status, education, employment status, and income). Differences between beneficiaries and non-beneficiaries of the ANRiN nutritional intervention were examined using chi-square tests for categorical variables. Independent *t*-tests were applied to compare mean scores for KAP related to IYCF. These scores were derived from the sum of correct responses to domain-specific questions, with knowledge and practice categorized as “adequate” or “inadequate,” and attitudes classified as “positive” or “negative” (33, 35).

To account for the multistage sampling design, design effects were calculated for each outcome. The design effect for knowledge (prevalence 21.1%) was 1.08, with an intra-cluster correlation coefficient (ICC) of 0.014, indicating minimal clustering effect on variance estimates. Assumptions for independent *t*-tests were checked using Shapiro-Wilk test for normality (*p* > 0.05 for all groups) and Levene's test for homogeneity of variance (*p* > 0.05 for all comparisons). For chi-square tests, expected cell frequencies were confirmed to be ≥5 in at least 80% of cells.

To identify predictors of each outcome, logistic regression analyses were conducted separately for knowledge, attitude, and practice, stratified by beneficiary status (beneficiaries vs. non-beneficiaries). In the first stage, univariable logistic regression was performed to calculate crude odds ratios (CORs) for potential predictors, including sociodemographic characteristics and exposure to healthcare information. Variables with *p* < 0.25 were considered for entry into the multivariable logistic regression models. Forward and backward likelihood ratio (LR) selection methods were applied, and the final model was estimated using the enter method to obtain adjusted odds ratios (AORs) with 95% confidence intervals. Sample size adequacy for regression models was assessed using the events per variable (EPV) criterion. For the knowledge model among non-beneficiaries (50 adequate knowledge cases, 5 predictors), EPV was 10.0; among beneficiaries (56 adequate knowledge cases, 5 predictors), EPV was 11.2, meeting the minimum recommended threshold of 10. Collinearity was assessed using variance inflation factors (VIF), with VIF > 10 considered indicative of problematic collinearity. Marital status and economic status were excluded from the final model due to collinearity (VIF > 10 for both variables). Receiver operating characteristic (ROC) curve analysis was performed to test the predictive validity of the regression models. The area under the curve (AUC) for the knowledge model among non-beneficiaries was 0.71 (95% CI: 0.64–0.78, *p* < 0.05) and among beneficiaries was 0.73 (95% CI: 0.67–0.79, *p* < 0.05), indicating acceptable model discrimination. Similar AUC values were observed for the attitude and practice models, confirming adequate model fit. Statistical significance was set at *p* < 0.05 for all analyses.

#### 2.8.2 Qualitative data analysis

Qualitative data analysis was guided by Braun and Clarke's thematic analysis framework (32). A combination of deductive and inductive (open coding) approaches was used to identify themes (36). The FGDs and KIIs recordings were professionally transcribed by a transcription service (Scribie.com) and subsequently cross-checked for accuracy and completeness by the research team.

Thematic analysis followed the six-phase framework of Braun and Clarke (32): (i) familiarization with the data, (ii) generating initial codes, (iii) searching for themes, (iv) reviewing themes, (v) defining and naming themes, and (vi) producing the report. Two independent researchers (PU and PO) coded a subset of transcripts (20%) to establish inter-coder reliability (Cohen's κ = 0.82). Discrepancies were resolved through consensus discussions. Initial coding was conducted using open coding techniques, followed by axial coding to identify relationships between codes. Themes were developed iteratively through constant comparison across data sources. Data saturation was confirmed when no new themes emerged from three consecutive transcripts. Member checking was conducted with 10 participants who reviewed and validated the identified themes. Thematic analysis was conducted manually and supplemented with code-based analysis to identify recurrent patterns and themes across data sources, providing a nuanced understanding of the contextual factors shaping IYCF behaviors.

## 3 Results

### 3.1 Quantitative findings

#### 3.1.1 Participant characteristics

A total of 503 mothers/caregivers consented to participate. Table 1 compares the sociodemographic characteristics of beneficiaries and non-beneficiaries. The majority of participants were aged over 30 years (58.4%), with no significant age difference between groups (*p* = 0.238). Beneficiaries were more likely to be married (94.3% vs. 84.0%, *p* < 0.001), live in larger households (51.3% vs. 46.5% with 4–6 members, *p* = 0.038), and have lower educational attainment (32.0% vs. 24.1% with primary education), though educational differences were not statistically significant (*p* = 0.163). Both groups were predominantly low-income earners (< NGN 50,000: 79.4% of beneficiaries vs. 84.0% of non-beneficiaries, *p* = 0.201).

**Table 1:** Comparison of the sociodemographic characteristics of beneficiaries and non-beneficiaries.

Variables	Categories	Yes (%)	No (%)	Total	χ^2^	*p*-value
Age (years)	Less than 30	125 (39.6)	84 (44.9)	209 (41.6)	1.391	0.238
	Greater than 30	191 (60.4)	103 (55.1)	294 (58.4)		
Marital status	Single	5 (1.6)	10 (5.3)	15 (3.0)	**14.731**	**< 0.001** ^*^
	Married	298 (94.3)	157 (84.0)	455 (90.5)		
	Divorced/Widowed	13 (4.1)	20 (10.7)	33 (6.6)		
Number of children	One	42 (13.3)	37 (19.8)	79 (15.7)	3.756	0.289
	Two	59 (18.7)	33 (17.6)	92 (18.3)		
	Three	79 (25.0)	43 (23.0)	122 (24.3)		
	4 and above	136 (43.0)	74 (39.6)	210 (41.7)		
Education level	No formal education	28 (8.9)	19 (10.2)	47 (9.3)	5.117	0.163
	Primary education	101 (32.0)	45 (24.1)	146 (29.0)		
	Secondary education	131 (41.5)	94 (50.3)	225 (44.7)		
	Tertiary education	56 (17.7)	29 (15.5)	85 (16.9)		
Occupation	Employed	175 (55.4)	112 (59.9)	287 (57.1)	0.977	0.323
	Unemployed	141 (44.6)	75 (40.1)	216 (42.9)		
Household size	1 to 3 members	38 (12.0)	37 (19.8)	75 (14.9)	**8.418**	**0.038** ^*^
	4 to 6 members	162 (51.3)	87 (46.5)	249 (49.5)		
	7 to 9 members	64 (20.3)	43 (23.0)	107 (21.3)		
	10 and above members	52 (16.4)	20 (10.7)	72 (14.3)		
Economic status	Low income (Less than NGN 50,000)	251 (79.4)	157 (84.0)	408 (81.1)	3.204	0.201
	Middle income (NGN 50,000–NGN 150,000)	64 (20.3)	28 (15.0)	92 (18.3)		
	High income (More than NGN 150,000)	1 (0.3)	2 (1.1)	3 (0.6)		

Bold with ^*^ indicates statistical significance at *p* < 0.05.

#### 3.1.2 Mothers' knowledge of IYCF

Table 2 presents the proportion of mothers with correct responses to individual knowledge items. While most mothers understood the timing of complementary feeding introduction (91%) and EBF (87%), significant gaps existed in knowledge of vitamin A benefits (31%) and iodine importance (8%). Overall, only 21% achieved an adequate knowledge score (≥70%).

**Table 2:** Proportion of mothers/caregivers with correct responses to knowledge items on IYCF.

Knowledge item	*N* (%) of correct answers
Correct answers for start time of breastfeeding	280 (56)
Correct answers for time of exclusive breastfeeding	438 (87)
Correct answers for benefits of breastfeeding	228 (45)
Correct answers for child age for complementary food introduction	456 (91)
Correct answer for frequency of complementary feeding 6–8 months	316 (63)
Correct answer for frequency of complementary feeding 9–23 months	217 (43)
Correct answer for balanced diet definition	385 (77)
Correct answer for groups of food given to child	69 (14)
Correct answer for benefits of Vitamin A	157 (31)
Correct answers for food rich in Iron	163 (32)
Correct answer for importance of Iodine	41 (8)
Knowledge score	
Adequate (≥70%)	106 (21)
Inadequate (< 70%)	397 (79)

#### 3.1.3 Mothers' attitudes toward IYCF

As shown in Table 3, most mothers (91%) demonstrated positive attitudes toward IYCF. The majority believed breastfeeding is better than formula feeding (86%), considered 6 months appropriate for introducing solid foods (82%), and recognized the importance of balanced nutrition (95%) and nutrition for child development (95%). Overall, 91% achieved a positive attitude score, while 9% had a negative attitude score.

**Table 3:** Distribution of statements on mothers' attitudes toward IYCF.

Statement	Category	*N* (%)
Between formula feeding and breastfeeding, which do you believe is better?	Breastfeeding	431 (86)
	Formula feeding	72 (14)
What is your opinion on introducing solid foods at 6 months?	Good/Appropriate	414 (82)
	Not good/Not sure	89 (18)
How important do you think it is to provide a balanced diet for a child?	Important	479 (95)
	Not important/Not sure	24 (5)
How important do you think good nutrition is for a child's overall development?	Important	476 (95)
	Not important/Not sure	27 (5)
Attitude score		
Positive (≥70%)	460 (91)	
Negative (< 70%)	43 (9)	

#### 3.1.4 Mothers' practices of IYCF

Table 4 presents the proportion of mothers reporting good practices. While nearly all mothers washed hands before feeding (99%) and ensured their child was up-to-date with immunizations (84%), fewer consistently engaged in regular health check-ups (16%), EBF for 6 months (84%), and frequent inclusion of fruits and vegetables in the child's diet (23%). Overall, 56% achieved a good practice score (≥70%).

**Table 4:** Distribution of mothers' practices on IYCF.

Practice item	*N* (%) of correct responses
Do you wash hands before feeding the child?	496 (99)
How often do you wash your hands before feeding your child?	397 (79)
How often do you take your child for health check-ups?	78 (16)
Is your child up-to-date with immunizations?	424 (84)
What is your breastfeeding practice score?	315 (63)
Was your child exclusively breastfed for the first 6 months?	424 (84)
During exclusive breastfeeding, how often do you breastfeed your child in a day?	374 (74)
Outside exclusive breastfeeding, how often do you breastfeed your child in a day?	303 (60)
Do you give your child vitamin supplements?	453 (90)
How often do you include fruits and vegetables in your child's diet?	117 (23)
Practice score	
Good (≥70%)	280 (56)
Poor (< 70%)	223 (44)

#### 3.1.5 Overall KAP mean scores

As shown in Table 5, there was no significant difference in knowledge scores between beneficiaries and non-beneficiaries (*p* = 0.715). However, non-beneficiaries demonstrated significantly higher attitude scores (*p* = 0.012), while beneficiaries exhibited significantly better practice scores (*p* < 0.001).

**Table 5:** Overall mean KAP score between beneficiaries and non-beneficiaries.

Variable	ANRiN beneficiary	*N*	Mean (SD)	*t*-value	*p*-value
Knowledge score	Yes	316	5.44 (2.16)	−0.365	0.715
	No	187	5.51 (2.21)		
Attitude score	Yes	316	3.52 (0.77)	−2.531	**0.012** ^*^
	No	187	3.68 (0.64)		
Practice score	Yes	316	6.95 (1.42)	4.494	**< 0.001** ^*^
	No	187	6.34 (1.57)		

Bold with ^*^ indicates statistical significance at *p* < 0.05.

#### 3.1.6 Predictors of knowledge

As shown in Table 6, predictors of IYCF knowledge differed between beneficiaries and non-beneficiaries. Among non-beneficiaries, tertiary education was significantly associated with adequate knowledge (AOR = 0.048, 95% CI: 0.010–0.238, *p* < 0.001), meaning mothers with tertiary education had 95.2% lower odds of inadequate knowledge (i.e., significantly higher odds of adequate knowledge) compared to those with primary/no formal education. Parity was also a significant predictor (*p* = 0.001), and employment was associated with better knowledge (AOR = 1.33, 95% CI: 1.06–1.67, *p* = 0.014). Among beneficiaries, tertiary education (AOR = 0.052, 95% CI: 0.010–0.271, *p* = 0.001) was significantly associated with adequate knowledge, healthcare provider information (AOR = 5.07, 95% CI: 2.34–10.99, *p* < 0.001) was the strongest predictor, and household size ≥10 members (AOR = 3.21, 95% CI: 1.22–8.45, *p* = 0.018) was also a significant predictor.

**Table 6:** Sociodemographic predictors of mothers' knowledge of IYCF.

Variables	Category	Non-beneficiaries (*n* = 187)			Beneficiaries (*n* = 316)		
		Adequate *n* (%)	Inadequate *n* (%)	AOR (95% CI)	Adequate *n* (%)	Inadequate *n* (%)	AOR (95% CI)
Age (years)	< 30 (Ref)	15 (17.9)	69 (82.1)	1.00	21 (16.8)	104 (83.2)	1.00
	≥30	35 (34.0)	68 (66.0)	0.81 (0.38–1.72)	35 (18.3)	156 (81.7)	0.72 (0.34–1.52)
Education level	Primary/No formal (Ref)	6 (9.4)	58 (90.6)	1.00	2 (1.2)	119 (98.8)	1.00
	Secondary	19 (20.2)	75 (79.8)	0.36 (0.11–1.18)	26 (19.8)	105 (80.2)	0.42 (0.14–1.26)
	Tertiary	25 (86.2)	4 (13.8)	**0.048 (0.010–0.238)** ^*^	28 (50.0)	28 (50.0)	**0.052 (0.010–0.271)** ^*^
Occupation	Unemployed (Ref)	28 (37.3)	47 (62.7)	1.00	20 (14.2)	121 (85.8)	1.00
	Employed	22 (19.6)	90 (80.4)	**1.33 (1.06–1.67)** ^*^	36 (25.7)	104 (74.3)	0.91 (0.47–1.77)
Household size	1–3 (Ref)	11 (29.7)	26 (70.3)	1.00	11 (28.9)	27 (71.1)	1.00
	4–6	27 (31.0)	60 (69.0)	0.87 (0.29–2.65)	43 (26.5)	119 (73.5)	0.69 (0.28–1.72)
	7–9	6 (14.0)	37 (86.0)	0.48 (0.10–2.31)	4 (6.3)	60 (93.8)	0.45 (0.12–1.72)
	≥10	6 (30.0)	14 (70.0)	0.68 (0.14–3.24)	12 (23.1)	40 (76.9)	**3.21 (1.22–8.45)** ^*^
Healthcare provider information	No (Ref)	10 (16.1)	52 (83.9)	1.00	13 (10.2)	115 (89.8)	1.00
	Yes	40 (32.0)	85 (68.0)	1.45 (0.68–3.10)	43 (22.9)	145 (77.1)	**5.07 (2.34–10.99)** ^*^

AOR < 1 indicates lower odds of inadequate knowledge (i.e., adequate knowledge); Marital status and economic status were excluded from the final model due to collinearity; Model adequacy (AUC): Non-beneficiaries = 0.71 (95% CI: 0.64–0.78); Beneficiaries = 0.73 (95% CI: 0.67–0.79); Bold with ^*^ indicates statistical significance at *p* < 0.05.

#### 3.1.7 Predictors of attitudes

As shown in Table 7, maternal education was a significant predictor of positive attitudes in both groups. Among non-beneficiaries, secondary education (AOR = 0.153, 95% CI: 0.056–0.419, *p* < 0.001) and tertiary education (AOR = 0.106, 95% CI: 0.021–0.536, *p* = 0.007) were significantly associated with positive attitudes, indicating 84.7% and 89.4% lower odds of negative attitudes (i.e., higher odds of positive attitudes), respectively. Among beneficiaries, secondary education (AOR = 0.22, 95% CI: 0.07–0.68, *p* = 0.009) and healthcare provider information (AOR = 2.88, 95% CI: 1.19–6.96, *p* = 0.019) were significant predictors. Employment was not statistically significant in either group.

**Table 7:** Sociodemographic predictors of mothers' attitudes toward IYCF.

Variables	Category	Non-beneficiaries (*n* = 187)			Beneficiaries (*n* = 316)		
		Positive *n* (%)	Negative *n* (%)	AOR (95% CI)	Positive *n* (%)	Negative *n* (%)	AOR (95% CI)
Education level	Primary/No formal (Ref)	53 (82.8)	11 (17.2)	1.00	108 (83.7)	21 (16.3)	1.00
	Secondary	86 (96.6)	3 (3.4)	**0.153 (0.056–0.419)** ^*^	122 (93.1)	9 (6.9)	**0.22 (0.07–0.68)** ^*^
	Tertiary	28 (96.6)	1 (3.4)	**0.106 (0.021–0.536)** ^*^	55 (98.2)	1 (1.8)	0.41 (0.09–1.86)
Occupation	Unemployed (Ref)	69 (92.0)	6 (8.0)	1.00	126 (89.4)	15 (10.6)	1.00
	Employed	98 (87.5)	14 (12.5)	1.60 (0.79–3.22)	159 (91.4)	15 (8.6)	1.48 (0.68–3.22)
Healthcare provider information	No (Ref)	57 (91.9)	5 (8.1)	1.00	104 (81.3)	24 (18.7)	1.00
	Yes	112 (89.6)	13 (10.4)	1.32 (0.54–3.22)	182 (93.8)	12 (6.2)	**2.88 (1.19–6.96)** ^*^

AOR < 1 indicates lower odds of negative attitude (i.e., positive attitude); Marital status and economic status were excluded from the final model due to collinearity; Bold with ^*^ indicates statistical significance at *p* < 0.05.

#### 3.1.8 Predictors of practices

As shown in Table 8, predictors of IYCF practices differed between beneficiaries and non-beneficiaries. Among non-beneficiaries, tertiary education (AOR = 0.171, 95% CI: 0.075–0.389, *p* < 0.001) was significantly associated with good practices (i.e., 82.9% lower odds of poor practice), employment (AOR = 1.94, 95% CI: 1.13–3.31, *p* = 0.016) was associated with better practices, and household size of 4–6 members (AOR = 2.79, 95% CI: 1.28–6.11, *p* = 0.010) predicted good practices. Among beneficiaries, tertiary education (AOR = 0.19, 95% CI: 0.07–0.52, *p* = 0.001) was significantly associated with good practices, and healthcare provider information (AOR = 2.34, 95% CI: 1.11–4.92, *p* = 0.025) was a significant predictor, while employment was not significant.

**Table 8:** Sociodemographic predictors of mothers' IYCF practices.

Variables	Category	Non-beneficiaries (*n* = 187)			Beneficiaries (*n* = 316)		
		Good *n* (%)	Poor *n* (%)	AOR (95% CI)	Good *n* (%)	Poor *n* (%)	AOR (95% CI)
Education level	Primary/No formal (Ref)	26 (40.6)	38 (59.4)	1.00	62 (48.1)	67 (51.9)	1.00
	Secondary	47 (50.0)	47 (50.0)	0.68 (0.35–1.33)	77 (58.8)	54 (41.2)	0.52 (0.25–1.08)
	Tertiary	25 (86.2)	4 (13.8)	**0.171 (0.075–0.389)** ^*^	43 (76.8)	13 (23.2)	**0.19 (0.07–0.52)** ^*^
Occupation	Unemployed (Ref)	41 (54.7)	34 (45.3)	1.00	68 (48.2)	73 (51.8)	1.00
	Employed	57 (50.9)	55 (49.1)	**1.94 (1.13–3.31)** ^*^	114 (65.5)	60 (34.5)	1.34 (0.77–2.31)
Household size	1–3 (Ref)	16 (43.2)	21 (56.8)	1.00	24 (63.2)	14 (36.8)	1.00
	4–6	55 (63.2)	32 (36.8)	**2.79 (1.28–6.11)** ^*^	95 (58.6)	67 (41.4)	0.86 (0.38–1.94)
	7–9	17 (39.5)	26 (60.5)	0.95 (0.39–2.31)	39 (60.9)	25 (39.1)	1.06 (0.43–2.62)
	≥10	10 (50.0)	10 (50.0)	1.12 (0.39–3.21)	24 (46.2)	28 (53.8)	0.52 (0.20–1.35)
Healthcare provider information	No (Ref)	38 (61.3)	24 (38.7)	1.00	62 (48.4)	66 (51.6)	1.00
	Yes	62 (49.6)	63 (50.4)	0.93 (0.53–1.63)	120 (61.9)	74 (38.1)	**2.34 (1.11–4.92)** ^*^

AOR < 1 indicates lower odds of poor practice (i.e., good feeding practices); Marital status and economic status were excluded from the final model due to multicollinearity; Bold with ^*^ indicates statistical significance at *p* < 0.05.

### 3.2 Qualitative findings

The qualitative findings reveal contextual barriers that directly explain the quantitative KAP gaps in Plateau State.

#### 3.2.1 Barriers to practicing good feeding despite awareness

Both beneficiaries and non-beneficiaries mentioned food insecurity, lack of time, and economic hardship as significant impediments to adopting recommended feeding practices either from CHWs for beneficiaries, or from health facilities for non-beneficiaries.

“*We know that it is important for a child to eat balanced food because they are very young and need it to grow, but most of us cannot afford it every time. Baby food is very expensive now”* (FGD 1/Beneficiary 1)

“*Sometimes, I only have time to cook once a day, so I give what is available and then give my child tom brown”* (FGD 2/Non-beneficiary 1)

Some respondents noted that older family members discouraged them from modern feeding practices, favoring traditional beliefs.

“*My mother-in-law said giving water to a baby less than six months is necessary, that the baby also needs water to grow well. She did not agree with giving only breastfeeding”* (FGD 3/Beneficiary 2)

“*There is a common saying in my place, they say that if you feed your child eggs early, they will become thieves. So, I feed my child properly with breastmilk”* (FGD 4/Non-beneficiary 2)

#### 3.2.2 Influence and impact of ANRiN food demonstrations

Beneficiaries cited that food demonstrations gave them tangible skills, particularly meal planning using affordable local ingredients.

“*[...] now I know how to make pap with groundnut and soya beans. It fills the child better”* (FGD 1/Beneficiary 3)

“*[...] I can now make tom brown, use the sachet powder [MNP] and use our local food product and combine them well to make it rich to feed our children”* (FGD 3/Beneficiary 4)

Despite skill acquisition, some struggled to recall or replicate demonstrations.

“*I forgot how they mixed the MNP with food. Maybe they should give us pictures or booklets in our local language that we can look at”* (FGD 5/Beneficiary 5)

“*Most times I forget to use the tom brown and the other one, MNP. I just make food and give my child. It is when I come across them in the house that I use them”* (FGD 1/Beneficiary 6)

Some mothers recommended follow-ups and refresher sessions to reinforce learning.

“*They should come more often or call us in small groups after the demonstration day. It will help us to do reminders among ourselves and bond as friends and reach each other to share experience”* (FGD 2/Beneficiary 7)

“*When they visit, we get very excited; but we are always addressed in large group. It can be very good if they do it as group. We want them to visit more and more also”* (FGD 4/Beneficiary 8)

#### 3.2.3 Male involvement and household decision-making

Across groups, men were rarely involved in ANRiN activities or feeding decisions.

“*My husband says it is a woman's duty. He does not attend the sessions or help with the feeding. But he uses his bike to take me to the centre”* (FGD 5/Beneficiary 9)

In some households, mothers expressed they could not act independently on what they learned.

“*Sometimes I want to buy something better for the baby, but he says no money. So, I will just manage with what I can get by myself”* (FGD 6/Non-beneficiary 3)

#### 3.2.4 Feedback on ANRiN delivery and suggestions for improvement

Demonstrations and visuals were seen as most effective, especially among low-literacy participants.

“*When they show us how to cook, we remember more. But talking alone is not enough”* (FGD 1/Beneficiary 10)

Mothers emphasized the need for food supplements or small cash transfers to enable practice.

“*Even if we learn, without money or food at home, we cannot do anything”* (FGD 3/Beneficiary 11)

Non-beneficiaries expressed interest in participating, citing a lack of awareness or exclusion.

“*I heard about ANRiN, but they never came to our side, maybe we are too far. Also, some of us did not understand what the program is about”* (FGD 7/Non-beneficiary 4)

#### 3.2.5 Stakeholders' perspectives on implementation challenges and opportunities

Health workers described overload and limited ability to do follow-up or in-depth counseling.

“*Sometimes we see 50 women in one day, and there is no time to check if all of them understand”* (KII/Stakeholder 1)

Younger mothers and those in hard-to-reach areas often missed out due to mobility or access issues.

“*We need more outreach; some young mothers are not even aware of the program”* (KII/Stakeholder 2)

Stakeholders advocated combining education with cash transfers and tailoring sessions for low-literacy mothers.

“*Some women understand better with songs and role plays. Also, little stipends could improve attendance”* (KII/Stakeholder 3)

Limited tracking of behavior change beyond session attendance was identified.

“*We don't know if they're practicing at home. More follow-up or caregiver reports could help”* (KII/Stakeholder 4)

## 4 Discussion

This study assessed the KAP of infant and young child feeding and their sociodemographic predictors among mothers of children aged 6–23 months in Plateau State, Nigeria, with access to the ANRiN nutritional intervention as a comparator. To our knowledge, this is one of the first studies to examine how participation in a large-scale nutritional program such as ANRiN is associated with maternal KAP of IYCF in this context.

### 4.1 Sociodemographic characteristics and program participation

Program participation was associated with specific sociodemographic characteristics. Women residing in larger households (4–6 members) were more likely to be beneficiaries than those in smaller households. This likely reflects ANRiN's targeting of households with higher economic vulnerability and greater reliance on social support (11, 37). Households with more dependents experience greater food insecurity and nutritional stress, making them priority targets for community-based nutrition interventions. Marital status was also significant, with married women more often being beneficiaries, similar to findings from Ghana (22) and consistent with evidence from Kwara State where over half of beneficiaries were married (28). The finding that married women were more often beneficiaries aligns with evidence suggesting that married women may benefit from stronger social support networks that facilitate program engagement (38). Spousal support, both emotional and instrumental (such as transportation to health facilities), may enhance women's ability to attend counseling sessions and adhere to recommended feeding practices.

The finding that beneficiaries had lower educational attainment suggests that the ANRiN intervention is reaching populations with fewer formal educational opportunities, which are often more vulnerable to malnutrition. This is consistent with reports that undernutrition is more prevalent in rural and less-educated populations in Nigeria (15). In the present study, household size was the most significant sociodemographic factor distinguishing beneficiaries from non-beneficiaries. Women living in larger households were more likely to be beneficiaries, indicating that the programme supports families with multiple dependents where nutritional needs are more pronounced and resources may be stretched thinner.

### 4.2 Patterns and gaps in knowledge, attitudes, and practices

The study identified substantial knowledge gaps in infant and young child feeding. Although most respondents understood the timing of complementary feeding and the importance of exclusive breastfeeding, few demonstrated adequate knowledge of micronutrients such as vitamin A and iodine. Only 21% achieved adequate nutrition knowledge, which is considerably lower than the 31–89% reported in other studies (33, 35, 39–42), but comparable to studies suggesting limited caregiver awareness of vital micronutrients (43–46). Contributing factors may include insufficient nutrition education, limited engagement by health workers, and prevailing socioeconomic challenges (47–49).

Interestingly, no significant difference in knowledge scores was observed between beneficiaries and non-beneficiaries. This may reflect the broader IYCF focus compared to EBF-specific knowledge in other studies, or variations in program implementation intensity. It may also indicate that non-beneficiaries in Plateau were exposed to nutrition information through other channels (media, other programs, community networks), resulting in knowledge diffusion or spillover effects within communities (22). This could attenuate observable differences between the groups, potentially underestimating the program's true association with improved knowledge outcomes.

Attitudes toward IYCF were generally positive, with 91% of mothers demonstrating positive attitudes. While beneficiaries and non-beneficiaries both showed high proportions of positive attitudes, non-beneficiaries demonstrated significantly higher attitude scores than beneficiaries. This may reflect social or cultural reinforcement outside formal programs (38, 50). The proportion of mothers with positive attitudes surpasses findings from Ethiopia (76%), Sierra Leone (59%), and Rwanda (87%) (51–53), though it was lower than the 98.4% reported in Kenya (54). The high proportion of positive attitudes despite low knowledge scores suggests that attitudes may be shaped by cultural norms and social desirability rather than accurate knowledge, a phenomenon observed in other studies where respondents express favorable views toward health behaviors without corresponding understanding of the underlying principles (55, 56).

Despite generally positive attitudes, only 56% demonstrated good feeding practices, revealing a clear knowledge-practice gap. Specific behaviors such as regular health check-ups (16%), exclusive breastfeeding for the first 6 months (84%), and frequent inclusion of fruits and vegetables in the child's diet (23%) were inconsistently followed. This discrepancy between knowledge and practice has been documented in previous studies, where high knowledge levels do not always translate into proper feeding practices (35, 47, 49). These findings reveal that while foundational practices like breastfeeding and hygiene are relatively well adopted, broader aspects of nutrition, particularly dietary diversity and preventive care, remain underutilized (35, 48, 57, 58).

The mechanism behind improved practice scores among beneficiaries is likely multi-faceted: first, the monthly counseling contacts provide repeated reinforcement of recommended practices, which is essential for behavior change (59). Second, the practical food demonstrations equip mothers with skills that address the “knowing-doing gap”, a phenomenon well-documented in health behavior literature where knowledge alone is insufficient without practical competency (60, 61). Third, the provision of micronutrient powders and other supplements reduces the financial barriers that often prevent mothers from implementing recommended practices (20). These findings are consistent with the Social Cognitive Theory, which posits that behavior change is achieved through a combination of knowledge acquisition, skill development, and self-efficacy building (62). Beneficiaries demonstrated significantly better practice scores than non-beneficiaries, suggesting that program participation is associated with improved adherence to recommended feeding behaviors. This aligns with findings from Oyo State where ANRiN implementation was associated with significantly improved EBF rates (26, 62), and from Ghana where beneficiaries showed 74.6% EBF rates (22).

### 4.3 Qualitative insights of the knowledge-practice gap

The qualitative findings provide important context for understanding this knowledge-practice gap. Beneficiaries and non-beneficiaries alike cited food insecurity, lack of time, and economic hardship as significant impediments to adopting recommended feeding practices. This highlights that even when mothers possess adequate knowledge, structural barriers such as food insecurity and economic hardship prevent them from translating this knowledge into practice, a finding consistent with studies in similar contexts (63–65).

The observed knowledge-practice gap in this study can be further understood through the lens of the Integrated Behavioral Model for Water, Sanitation, and Hygiene (IBM-WASH), adapted here to nutrition context. This framework posits that behavior change is influenced by multiple levels of factors: individual (e.g., knowledge, attitudes, self-efficacy), interpersonal (e.g., family support, social norms), and structural (e.g., food access, economic constraints) (66, 67). In our study, despite adequate knowledge and positive attitudes at the individual level, structural barriers, particularly food insecurity and poverty, created an enabling environment that undermined translation of knowledge into practice (68).

This phenomenon has been termed the “intention-behavior gap” in health behavior literature (69, 70). According to the Theory of Planned Behavior, intentions are shaped by attitudes, subjective norms, and perceived behavioral control. Our qualitative findings suggest that while attitudes and subjective norms were favorable, perceived behavioral control was low due to economic constraints, resulting in weak intention-behavior concordance (71). This explains why mothers with knowledge did not always practice recommended behaviors; they lacked the necessary resources to do so.

Furthermore, the absence of male involvement in feeding decisions identified in our qualitative findings represents a gap at the interpersonal level. Research from similar contexts has shown that male partners often control household resources, limiting women's agency in nutrition-related decisions (38, 63). This finding is consistent with studies from Ethiopia and Bangladesh where women's decision-making autonomy was a stronger predictor of child feeding practices than nutrition knowledge alone (72, 73).

Additionally, some respondents noted that older family members discouraged them from modern feeding practices, favoring traditional beliefs, underscoring the importance of engaging broader household members in behavior-change interventions.

Beneficiaries reported that food demonstrations gave them tangible skills, particularly meal planning using affordable local ingredients. However, despite skill acquisition, some struggled to recall or replicate demonstrations, highlighting the need for simplified pictorial job aids in local languages and more frequent follow-up visits. These findings suggest that while the program provides valuable practical training, retention and reinforcement remain challenges that need to be addressed.

### 4.4 Predictors of KAP outcomes

The sociodemographic predictors identified in this study align with previous research while revealing important differences between beneficiaries and non-beneficiaries.

Education was consistently the strongest predictor across all outcomes and both groups. Mothers with tertiary education were substantially more likely to have adequate knowledge, positive attitudes, and good practices compared with those with no formal education. This is consistent with studies by Iftikhar et al. (74), Tariqujjaman et al. (75), and Amugsi et al. (76), and mirrors findings from Kwara where tertiary education was significantly associated with good knowledge among both groups (28). Education enhances cognitive skills, health literacy, and access to nutrition information, all essential for making informed feeding decisions (74, 75). Women with formal education are better equipped to process and retain health information, critically evaluate feeding recommendations, and navigate health systems to access nutrition services. The strong association between tertiary education and KAP outcomes across both groups underscores the importance of maternal education as a fundamental determinant of child nutrition, consistent with the findings of Amugsi et al. (76) in Ghana and the broader literature on women's education and child health outcomes.

Parity significantly influenced knowledge among non-beneficiaries, with mothers of one or two children demonstrating better knowledge than those with four or more children. This aligns with findings by Iftikhar et al. (74), Jensen et al. (77), and Batura et al. (78), suggesting that as the number of children increases, mothers may have less time and resources to seek and retain nutritional information.

Healthcare provider information was strongly associated with knowledge and attitudes among beneficiaries. This highlights the significant role of healthcare providers as trusted sources of nutrition information, consistent with findings from Kwara where providers emerged as the strongest predictor of good knowledge among beneficiaries (28), and with other studies (79, 80). This finding underscores the importance of strengthening provider counseling services and ensuring that community health workers are adequately trained and resourced to deliver effective nutrition education. The trust that mothers place in healthcare providers may enhance the credibility and acceptability of nutrition messages, facilitating behavior change.

Employment status showed distinct patterns: among non-beneficiaries, being employed was associated with better practices, likely reflecting economic stability enabling access to nutritious foods and healthcare (81, 82). Employed women have greater financial resources to purchase nutrient-dense foods, pay for transportation to health facilities, and afford supplements when needed. However, among beneficiaries, employment was not a significant predictor, suggesting that program participation may compensate for employment-related barriers through direct service provision, including the supply of micronutrient supplements and counseling that reduces the need for out-of-pocket expenditure on nutrition-related items.

Household size predicted better practices among non-beneficiaries, possibly reflecting resource pooling and shared childcare responsibilities in larger households. Among beneficiaries, healthcare provider information was the strongest practice predictor, aligning with evidence of the role of healthcare access and counseling in enabling feeding adherence (63–65).

### 4.5 Strengths and limitations

This study has several strengths. The use of a well-defined sample of both beneficiaries and non-beneficiaries, stratified multivariate analysis by beneficiary status, and the addition of a qualitative component allowed for nuanced understanding of relationships between sociodemographic characteristics and KAPs. The mixed-methods design provided rich contextual insights that quantitative data alone could not capture. The validated and reliable questionnaire (Cronbach's α: 0.78–0.82), rigorous data collection procedures using KoboCollect, and robust statistical analysis including ROC curve analysis to validate model fit enhance the credibility and reproducibility of the findings.

However, several limitations must be acknowledged. The cross-sectional design restricts causal interpretations. Self-reported data may be influenced by social desirability bias. Findings are specific to Plateau State and may lack generalizability. The sampling approach, selecting both beneficiaries and non-beneficiaries from the same intervention communities, carries potential for knowledge diffusion or spillover effects, which could attenuate observable differences between groups. The study focused on mothers/caregivers and did not include fathers or other family members who may influence feeding decisions. The qualitative component was limited to two LGAs and may not capture the full diversity of experiences across the state.

## 5 Conclusion

This study provides important insights into the knowledge, attitudes, and practices of mothers and caregivers regarding infant and young child feeding in Plateau State, Nigeria. The findings demonstrate that ANRiN participation is associated with enhanced feeding practices among beneficiaries (mean practice score 6.95 ± 1.42 vs. 6.34 ± 1.57 among non-beneficiaries, *p* < 0.001), particularly in rural and socioeconomically vulnerable populations. However, significant gaps remain in maternal nutritional knowledge, with only 21% achieving adequate knowledge scores, especially regarding micronutrients (only 31% knew benefits of vitamin A, 8% understood iodine importance), despite generally positive attitudes (91% positive). The translation of knowledge into sustained practice remains constrained by structural barriers, with only 56% achieving good feeding practices despite 91% positive attitudes, revealing a substantial knowledge-practice gap.

Distinct predictors between beneficiaries and non-beneficiaries suggest that tailored interventions accounting for these differential factors could strengthen IYCF program effectiveness. Education (tertiary education: AOR = 0.048–0.052, *p* < 0.01) and employment (AOR = 1.94, *p* = 0.016 among non-beneficiaries) were strong predictors of better practices, highlighting the central role of maternal empowerment. Healthcare provider information was the strongest predictor among beneficiaries (knowledge: AOR = 5.07, *p* < 0.001; practice: AOR = 2.34, *p* = 0.025), indicating the importance of strengthening provider counseling services. To bridge the knowledge-practice gap, future interventions should develop pictorial job aids in local languages, engage influential family members in community education, explore social protection measures like cash transfers, and expand nutrition education for low-literacy mothers.

Future initiatives should prioritize scalable, gender-inclusive frameworks that address individual and contextual determinants of feeding behavior. Longitudinal research is needed to ascertain causal impacts, and expanding studies across diverse regions would enhance generalizability. Integrating qualitative methods could provide deeper insights into cultural and social factors influencing IYCF practices.

## Data Availability

The original contributions presented in the study are included in the article/Supplementary material, further inquiries can be directed to the corresponding author.
