# Supplementary material for: Patterns and predictors of infant and young child feeding practices among mothers of children aged 6–23 months in Plateau State, Nigeria: a mixed-methods comparison of nutrition intervention beneficiaries and non-beneficiaries
Source: Front Nutr. 2026 Sep 30;13:1951497. doi: 10.3389/fnut.2026.1951497 (PMC13661402; doi:10.3389/fnut.2026.1951497)
Supplement: Supplementary file 1 [file Data_Sheet_1.pdf]

Assessment of maternal knowledge and attitude towards infant and young child nutrition and feeding practice in Plateau State, Nigeria

Survey Introduction

Introduce the purpose of the data collection to the participant and what it is all about

hh:mm

Consent form

Request for consent from the participant and ensure they sign the consent for with their name, signature and date

hh:mm

LGA

Select the right LGA

- ◯ Shendam LGA
- ◯ Mangu LGA

Ward (Shendam LGA)

- ◯ Shendam B
- ◯ Yelwa East

Community (Shendam B)

- ◯ Angwan Kabari (Shendam B)
- ◯ Anguwan Kwanan Dadi (Shendam B)

Community (Yelwa East)

- ◯ Angwan Jarawa (Yelwa East)
- ◯ Angwan Galadima (Yelwa East)

Ward (Mangu LGA)

- ◯ Mangu 1
- ◯ Mangu 2

Community (Mangu 1)

- ◯ Angwan Madaki (Mangu 1)
- ◯ Rampiya (Mangu 1)

Community (Mangu 2)

- ◯ Angwan Sarki (Mangu 2)
- ◯ Bungha Dawo (Mangu 2)

Name of Participant

Enter name of participant

Are you a beneficiary of the ANRiN project?

- ◯ Yes
- ◯ No

How old are you?

Select the age range of participant upon asking

- ◯ Under 20
- ◯ 20 to 29
- ◯ 30 to 39
- ◯ 40 to 49
- ◯ 50 and above

What is your marital status?

Select the marital status that apply to participant

- ◯ Single
- ◯ Married
- ◯ Divorced
- ◯ Widowed

How many children do you have?

Select number of children of participant upon asking

- ◯ 1
- ◯ 2
- ◯ 3
- ◯ 4 and above

What is your highest level of education?

Select the level of education of participant

- ◯ No formal education
- ◯ Primary education
- ◯ Secondary education
- ◯ Tertiary education

What is your source of information regarding child nutrition and breastfeeding practice?

- ◯ Health care providers (doctors, nurses, midwives)
- ◯ Family and friends
- ◯ Community health workers
- ◯ Internet resources (websites, social media)
- ◯ Books and magazines
- ◯ Television and radio programs
- ◯ Parenting classes or workshops
- ◯ Traditional knowledge or practices

What is your employment status?

- ◯ Unemployed
- ◯ Self-employed
- ◯ Employed

What is your household size?

- ◯ 1 to 3 members
- ◯ 4 to 6 members
- ◯ 7 to 9 members
- ◯ 10 and above members

What is your knowledge of nutrition?

- ◯ Poor (0 to 25%)
- ◯ Fair (26 to 50%)
- ◯ Good (51 to 75%)
- ◯ Excellent (76 to 100%)

What is your monthly income?

- ◯ Low income (Less than NGN 50,000)
- ◯ Middle income (NGN 50,000 - NGN 150,000)
- ◯ High income (More than NGN 150,000)

How old is your child?

- ◯ Below 6 months
- ◯ 6-11 months
- ◯ 1-2 years
- ◯ 3-4 years
- ◯ 5 years

What is your child's gender?

- ◯ Male
- ◯ Female

What was your child's birth weight?

- ◯ Less than 2.5 kg
- ◯ 2.5 - 3.5 kg
- ◯ More than 3.5 kg
- ◯ I do not remember

When did you start breastfeeding your child?

- ◯ Immediately after birth
- ◯ After a few hours
- ◯ Next day
- ◯ Some days later

How long should exclusive (i.e., only given breast milk, no other foods or liquids, not even water) breastfeeding continue?

- ◯ 0-3 months
- ◯ 4-5 months
- ◯ 6 months
- ◯ More than 6 months
- ◯ I don't know

What are the benefits of breastfeeding for the baby?

- ◯ Provides essential nutrients
- ◯ Boosts immunity (helps them fight diseases)
- ◯ Enhances bonding (creates a strong emotional connection)
- ◯ All of the above
- ◯ I don't know

At what age should complementary foods be introduced?

- ◯ Before 6 months
- ◯ At 6 months
- ◯ After 6 months
- ◯ I don't know

How often should complementary foods be given to a child aged 6-8 months?

- ◯ Once a day
- ◯ Twice a day
- ◯ Three times a day or more
- ◯ I don't know

How often should complementary foods be given to a child aged 9-23 months?

- ◯ Twice a day
- ◯ Three times a day
- ◯ Four times a day or more
- ◯ I don't know

What types of complementary foods should be given? (Select all that apply)

- ☐ Grains, roots, tubers (Corn, yam, cassava, cocoa yam, millets)
- ☐ Legumes (beans) and nuts
- ☐ Dairy products (Milk, Custard, Yoghurt, Fura da Nono, Butter, Oatmeal, Pap)
- ☐ Flesh foods (meat, fish, poultry, organs)
- ☐ Eggs
- ☐ Vitamin A-rich fruits and vegetables
- ☐ Others (specify)

Specify the others

____

How often should a young child be fed solid foods in addition to breast milk?

- ◯ Once a day
- ◯ Twice a day
- ◯ Three times a day

Do you know what a balanced diet means or includes?

- ◯ Yes
- ◯ No

Which of the following are essential nutrients for a child's growth? (Select all that apply)

- ☐ Proteins
- ☐ Carbohydrates
- ☐ Fats
- ☐ Vitamins
- ☐ Minerals

How many different food groups does your child eat in a day? (e.g., grains, fruits, vegetables, proteins, dairy foods)

- ◯ 1-2 groups
- ◯ 3-4 groups
- ◯ More than 4 groups
- ◯ I cannot tell

What are the benefits of vitamin A for children?

- ◯ Improves vision
- ◯ Strengthens immune system
- ◯ Promotes growth
- ◯ All of the above
- ◯ I don't know

Which foods are rich in iron and important for young children?

- ◯ Red meat
- ◯ Leafy greens
- ◯ Beans
- ◯ All of the above
- ◯ I don't know

What is the importance of iodine in a child's diet?

(Iodine is a mineral found in some foods such as salt)

- ◯ Prevents goiter
- ◯ Supports brain development
- ◯ Improves metabolism
- ◯ All of the above
- ◯ I don't know

How do you ensure the safety of the food given to your child? (Multiple answers possible)

- ☐ Washing hands before preparing food
- ☐ Washing food items before cooking
- ☐ Using clean utensils
- ☐ Storing food properly

Do you wash hands before feeding the child?

- ◯ Yes
- ◯ No

How often do you wash your hands before feeding your child?

- ◯ Always
- ◯ Sometimes
- ◯ Never

Why is it important to wash your hands before feeding your child? (Multiple responses)

- ☐ Prevents Spread of Germs
- ☐ Reduces Risk of Foodborne Illness
- ☐ Maintains Hygiene
- ☐ Prevents Contamination
- ☐ Promotes Overall Health

How often do you take your child for health check-ups?

- ◯ Monthly
- ◯ Quarterly
- ◯ Annually
- ◯ Never

Is your child up-to-date with immunizations?

- ◯ Yes
- ◯ No

Do you give your child any nutritional supplements (e.g., vitamins, minerals)?

- ◯ Yes
- ◯ No

Which of the following are signs of malnutrition in children? (Select all that apply)

- ☐ Weight loss
- ☐ Weakness
- ☐ Swelling in the body
- ☐ Hair loss
- ☐ Pale skin

Are there any health issues affecting your child's nutrition?

- ◯ Yes
- ◯ No

Which health issues is affecting your child's nutrition?

- ◯ Food allergies
- ◯ Stomach problems (constipation, diarrhea)
- ◯ Trouble digesting milk
- ◯ Gluten intolerance (celiac disease)
- ◯ Acid reflux
- ◯ Low iron (anemia)
- ◯ Vitamin deficiencies
- ◯ Frequent infections
- ◯ Poor appetite
- ◯ Chronic illnesses (asthma, diabetes)
- ◯ Growth problems

Between formula feeding and breastfeeding, which do you believe is better?

- ◯ Formula feeding
- ◯ Breastfeeding
- ◯ Both equally
- ◯ Not sure

How comfortable are you with breastfeeding in public?

- ◯ Very comfortable
- ◯ Somewhat comfortable
- ◯ Not comfortable

What is your opinion on introducing solid foods at 6 months?

- ◯ Very important
- ◯ Somewhat important
- ◯ Not important

How important do you think it is to provide a balanced diet for a child?

- ◯ Very important
- ◯ Somewhat important
- ◯ Not important

How important do you think good nutrition is for a child's overall development?

- ◯ Very important
- ◯ Somewhat important
- ◯ Not important

What challenges do you face in providing nutritious food to your child? (Multiple answers allowed)

- [ ] Financial constraints
- [ ] Lack of knowledge
- [ ] Lack of availability
- [ ] Cultural practices
- [ ] Others (specify)

Others (specify)

____

What is your breastfeeding practice score?

- ( ) Poor (0-40%) (1 to 2 times daily)
- ( ) Fair (41-70%) (3 to 5 times daily)
- ( ) Good (71-100%) (6 times and above daily)

Was your child exclusively (i.e., only given breast milk, no other foods or liquids, not even water) breastfed for the first 6 months?

- ( ) Yes
- ( ) No

During exclusive breastfeeding, how often do you breastfeed your child in a day?

- ( ) 1-2 times
- ( ) 3-5 times
- ( ) More than 5 times

Outside exclusive breastfeeding, how often do you breastfeed your child in a day?

- ( ) 1-2 times
- ( ) 3-5 times
- ( ) More than 5 times

What foods did you first introduce to your child? (Multiple answers allowed)

- [ ] Cereals (Cerelac, Pap, Oatmeal, Golden morn, Custard)
- [ ] Fruits (Oranges, Banana, Watermelon, Apple, Pear, Pineapple etc)
- [ ] Vegetables
- [ ] Proteins
- [ ] Carbohydrates (Garri, yam, etc)

Do you give your child vitamin supplements (e.g., Vitamin A, Vitamin D, Vitamin E, etc.)?

- ( ) Yes
- ( ) No

How often do you include fruits and vegetables in your child's diet?

- ◯ Daily
- ◯ Weekly
- ◯ Rarely

What are the main challenges you face in feeding your child?

- ☐ Lack of access to nutritious food
- ☐ Child's lack of appetite or picky eating
- ☐ Financial constraints
- ☐ Limited knowledge about proper nutrition
- ☐ Time constraints due to work or other commitments
- ☐ Health issues affecting my child's ability to eat
- ☐ Cultural or dietary restrictions
- ☐ Difficulty in preparing healthy meals
- ☐ Food allergies or intolerances
- ☐ Inconsistent feeding schedule

What support do you need to improve your child's nutrition?

- ☐ Access to affordable nutritious food
- ☐ Nutrition education and guidance
- ☐ Financial assistance or food vouchers
- ☐ Recipes and meal planning ideas
- ☐ Support from healthcare professionals (e.g., pediatricians, dietitians)
- ☐ Time management tips for preparing healthy meals
- ☐ Information on managing food allergies or intolerances
- ☐ Information on local resources and programs for child nutrition

Are there any traditional practices that influence how you feed your child?

- ☐ Cultural dietary restrictions
- ☐ Traditional weaning practices
- ☐ Use of traditional herbs and remedies
- ☐ Beliefs about certain foods and health
- ☐ Involvement of extended family in feeding decisions
- ☐ Rituals or ceremonies related to food
- ☐ Traditional methods of food preparation and cooking
- ☐ I do not follow any traditional practices

How do community beliefs affect your feeding practices?

- [ ] Influence of local dietary traditions
- [ ] Recommendations from community elders
- [ ] Pressure to adhere to communal norms
- [ ] Adoption of widely accepted feeding schedules
- [ ] Use of community-endorsed herbal remedies
- [ ] Avoidance of foods considered taboo
- [ ] Participation in communal feeding practices
- [ ] Incorporation of advice from local healers
- [ ] Reliance on communal support networks for food
- [ ] No significant influence from community beliefs
