# Supplementary material for: Patterns and predictors of infant and young child feeding practices among mothers of children aged 6–23 months in Plateau State, Nigeria: a mixed-methods comparison of nutrition intervention beneficiaries and non-beneficiaries
Source: Front Nutr. 2026 Sep 30;13:1951497. doi: 10.3389/fnut.2026.1951497 (PMC13661402; doi:10.3389/fnut.2026.1951497)
Supplement: Supplementary file 2 [file Data_Sheet_2.pdf]

FGD and KII Guides

Consent Statement

Good morning/afternoon. Thank you for agreeing to participate in this discussion. We are conducting this research to better understand the knowledge, attitudes, and practices regarding infant and young child feeding (IYCF) in your community, especially in relation to the ANRiN project. Your experiences, opinions, and insights are very important to us.

Participation in this discussion is entirely voluntary. You are free to choose not to answer any question and may stop the interview at any time without any consequence. There are no right or wrong answers—we are simply interested in your honest thoughts and experiences.

This session will be audio-recorded to help us accurately capture what is said, but your identity will remain confidential. Your name will not appear in any report or publication. We will treat all the information you provide with strict confidentiality and use it solely for research purposes.

If you have any questions, please feel free to ask. If you agree to participate and for us to record this session, we will proceed now.

[Pause for verbal consent. If agreed, continue:]

Thank you.

Focus Group Discussion (FGD) Guide

Participants: Mothers/Caregivers (Beneficiaries of ANRiN project intervention)

S/N	Questions with probing questions
1	Some mothers know about good feeding practices but struggle to follow them. What makes it hard to practice what you know?<br><br>***Probes***:<br>i. Can you share examples of advice you find difficult to follow? Why?<br>ii. How do limited resources (money, food, time) affect your ability to act on what you've learned?
2	How have ANRiN's food demonstrations sessions helped you? What's still unclear?<br><br>***Probes***:<br>i. Do you have anything that is not clear to you regarding food demonstration?<br>ii. Can explain any skill or knowledge you gained or learned from the ANRiN food demonstration sessions that you now use daily?<br>iii. What parts of the demonstrations were hard to remember or apply at home?
3	What traditions or family opinions influence how you feed your child?<br><br>***Probes***:<br>i. Have you faced any form of discouragement in trying to apply the learnings or knowledge gained by any family member or anyone? (Explain)<br>ii. How did it make you feel and you handle this?
4	Who makes the final decisions about what your child eats in your household?<br><br>***Probes***:<br>i. Can you describe a time when you wanted to feed your child differently but couldn't? What stopped you?<br>ii. How do male family members influence feeding practices? Are they involved in ANRiN activities?<br>iii. What would help get men more engaged in child nutrition?
5	If you could change one thing about how ANRiN teaches feeding practices, what would it be?<br><br>***Probes***:<br>i. What teaching methods work best for you? (demonstrations, pictures, group discussions) and why?<br>ii. How could the program better support mothers between visits?<br>iii. What additional resources would help you practice what you learn?
6	Is there anything else you would like to share about feeding your child or any ideas you have for how programs like ANRiN can better support mothers like you?

Focus Group Discussion (FGD) Guide

Participants: Mothers/Caregivers (Non-Beneficiaries of ANRiN Project)

S/N	Questions with probing questions
1	What do you know about proper infant and young child feeding (IYCF) practices, and what makes it hard to follow them?<br><br>***Probes***:<br><br>i. Can you share examples of feeding advice you've heard that's difficult to practice? Why?<br>ii. How do challenges like money, food access, or time affect how you feed your child?<br>iii. Have you heard of ANRiN's food demonstrations? If yes, why have not you participated?
2	What do you know about proper infant and young child feeding (IYCF) practices, and what makes it hard to follow them?<br><br>***Probes***:<br><br>i. Can you share examples of feeding advice you've heard that's difficult to practice? Why?<br>ii. How do challenges like money, food access, or time affect how you feed your child?<br>iii. Have you heard of ANRiN's food demonstrations? If yes, why have not you participated?
3	Where do you currently learn about child feeding practices?<br><br>***Probes***:<br><br>i. Who teaches you about child nutrition? (Health workers, family, media, etc.)<br>ii. What parts of this advice are easiest or hardest to follow? Why?<br>iii. How confident do you feel about the information you receive?
4	What traditions or family opinions shape how you feed your child?"<br><br>***Probes***:<br><br>i. Has anyone discouraged you from trying new feeding methods? How did you respond?<br>ii. How do male family members influence feeding decisions? Should they be more involved?
5	Who decides what your child eats in your household?<br><br>***Probes***:<br><br>i. Describe a time you couldn't feed your child as you wanted. What stopped you?<br>**ii.** What would help families work together better on child nutrition?
6	If a program like ANRiN wanted to help mothers like you, what should it include?<br><br>***Probes***:<br><br>i. What teaching methods would work best for you? (e.g., home visits, videos, community groups)<br>ii. What resources (e.g., food, money, tools) would make it easier to follow good feeding practices?<br>iii. How could programs better reach mothers who aren't currently participating?
6	Is there anything else you would like to share about your experiences with feeding your child or any suggestions you have for programs that could support mothers like you?

Key Informant Interview (KII) Guide

S/N	Questions with probing questions
1	Can you comment on any challenges you observed that do you think hindered mothers from applying ANRiN's lessons?<br>***Probes***:<br>i. How do time constraints or workload affect your counselling quality?<br>ii. How you do reach mothers in areas that are hard to reach, as well as the young mothers?
2	How could ANRiN's design better support behavior change and improvement in IYCN?
3	In what way do you think ANRiN program can be made more effective to support mother?<br>***Probes***:<br>i. Should cash transfers be paired with education? Why?<br>ii. How can we tailor programs for low-literacy mothers?
4	How does ANRiN currently track whether mothers are actually changing feeding practices?<br>***Probes***:<br>i. What indicators best show real behavior change versus just knowledge gain?<br>ii. How could the program better measure long-term impact?<br>iii. What data is currently being underutilized that could improve programming?
5	How does ANRiN's implementation compare to other nutrition programs you have worked with?<br>***Probes***:<br>i. What unique strengths does ANRiN have that should be preserved?<br>ii. What lessons from other programs could improve ANRiN?<br>iii. How does working with state partners affect implementation?
6	Is there anything else you would like to add about your experience with the ANRiN program or any recommendations you believe would help improve its impact and sustainability?
